# Patients with Type 2 Diabetes, Higher Blood Pressure, and Infrequent Fundus Examinations Have a Higher Risk of Sight-Threatening Retinopathy

**DOI:** 10.3390/jcm13092496

**Published:** 2024-04-24

**Authors:** Martina Tomić, Romano Vrabec, Spomenka Ljubić, Ingrid Prkačin, Tomislav Bulum

**Affiliations:** 1 Department of Ophthalmology, Vuk Vrhovac University Clinic for Diabetes, Endocrinology and Metabolic Diseases, Merkur University Hospital, 10000 Zagreb, Croatia; 2 Department of Diabetes and Endocrinology, Vuk Vrhovac University Clinic for Diabetes, Endocrinology and Metabolic Diseases, Merkur University Hospital, 10000 Zagreb, Croatia; 3 School of Medicine, University of Zagreb, Šalata 3, 10000 Zagreb, Croatia; 4 Department of Internal Medicine, Merkur University Hospital, 10000 Zagreb, Croatia

**Keywords:** diabetic retinopathy, fundus examination, systemic risk factors control, blood pressure, prevention

## Abstract

**Background:** Diabetic retinopathy (DR) is the most common cause of preventable blindness among working-age adults. This study aimed to evaluate the impact of the regularity of fundus examinations and risk factor control in patients with type 2 diabetes (T2DM) on the prevalence and severity of DR. **Methods:** One hundred and fifty-six T2DM patients were included in this cross-sectional study. **Results:** In this sample, the prevalence of DR was 46.2%. Patients with no DR mainly did not examine the fundus regularly, while most patients with mild/moderate nonproliferative DR (NPDR) underwent a fundus examination regularly. In 39.7% of patients, this was the first fundus examination due to diabetes, and 67% of them had sight-threatening DR (STDR). Diabetes duration (*p* = 0.007), poor glycemic control (HbA_1_c) (*p* = 0.006), higher systolic blood pressure (SBP) (*p* < 0.001), and diastolic blood pressure (DBP) (*p* = 0.002) were the main predictors of DR. However, the impact of SBP (AOR 1.07, *p* = 0.003) and DBP (AOR 1.13, *p* = 0.005) on DR development remained significant even after adjustment for diabetes duration and HbA_1_c. The DR prevalence was higher in patients with higher blood pressure (≥130/80 mmHg) than in those with target blood pressure (<130/80 mmHg) (*p* = 0.043). None of the patients with target blood pressure had STDR. The peaks in SBP and DBP were observed in T2DM with DR and the first fundus examination due to diabetes. **Conclusions:** In this T2DM sample, DR prevalence was very high and strongly related to blood pressure and a lack of regular fundus examinations. These results indicate the necessity of establishing systematic DR screening in routine diabetes care and targeting blood pressure levels according to T2DM guidelines.

## 1. Introduction

Diabetes is one of the fastest-growing global health emergencies of the 21st century, reaching alarming levels and projected to affect over 700 million people by 2045 [1]. As a primary driver of mortality worldwide, it was estimated to have caused over 6 million or 12.2% of global deaths in 2021 [1]. Besides the high rate of mortality and lower quality of life, diabetes and its complications also impose a significant economic impact on countries, health systems, and individuals with diabetes and their families. In 2021, global health expenditure due to diabetes was USD 966 billion, and the International Diabetes Federation (IDF) projects that total diabetes-related health expenditure will reach USD 1.05 trillion by 2045 [1].

Diabetic retinopathy (DR) is a common microvascular complication of diabetes and the leading cause of preventable blindness in the adult working population [2]. Several studies suggest that the prevalence of DR in patients with diabetes is approximately 22%, but most importantly, approximately 10% of patients with diabetes have a sight-threatening DR (STDR) such as proliferative DR (PDR) and/or diabetic macular edema (DME) [3]. STDR can often be prevented if DR is diagnosed, monitored, and treated in its earlier stage(s). The World Health Organization, in its global action plan, stressed the importance of the reduction of preventable blindness, including that related to diabetes. In recent years, the increasing intravitreal use of anti-vascular endothelial growth factor (anti-VEGF) agents and steroids resulted in a declining trend of new visual impairment and blindness due to diabetes [4,5]. However, the primary therapeutic strategies for preventing DR development and progression are good control of systemic risk factors and regular screening [6].

DR screening aims to detect initial changes in the retina that can be treated successfully, preventing the development of STDR and vision loss due to diabetes. The WHO recommends regular screening every one to two years if no abnormality is detected. Once DR is detected, the screening intervals are adjusted according to the DR’s severity and the patient’s systemic risk factors control. Patients with periodic and not regular DR screening represent a high-risk group with a greater probability of developing STDR in the meantime [7]. Some countries in the WHO European Region showed a declining trend of blindness related to diabetes due to coordinated public health education efforts, increased awareness, early detection using DR screening, sustained systemic risk factor control, and the availability of effective retinal treatment [8,9,10].

In the Republic of Croatia, there is no systemic DR screening program or registry of diabetic retinopathy and blindness due to diabetes [11]. Screening for DR is performed using dilated slit-lamp fundus examination only by ophthalmologists, mostly medical retina specialists; no other medical personnel (nurses, technicians, optometrists, etc.) are involved in the DR screening. In addition, no new technologies, such as automated grading, electronic data transfer systems (including telemedicine), artificial intelligence, national surveys, systems to monitor the screening frequency, or call–recall systems for those with long-standing diabetes and risk of DR, have been introduced into DR screening in Croatia.

Hyperglycemia, high blood pressure (BP), and dyslipidemia represent the most essential risk factors for DR. This study aimed to assess the impact of the regularity of fundus examinations and risk factor control on the prevalence and severity of DR in T2DM patients.

## 2. Materials and Methods

### 2.1. Study Design, Demographic Data, and Medical Records

This cross-sectional study was performed at the Department of Ophthalmology and Department of Diabetes and Endocrinology in Vuk Vrhovac University Clinic for Diabetes, Endocrinology, and Metabolic Diseases in Zagreb. A total of 156 T2DM patients attending both departments on the same day during the three months between 15 December 2020 and 15 March 2021 were included in the study. This study included patients over 18 years old with T2DM with a minimum of 1 year of disease duration. Those with advanced kidney and liver disease were excluded from the study. This study was conducted following the Declaration of Helsinki and was approved by the Hospital’s Ethics Committee. All study participants received written and oral information about the study and signed the written informed consent. 

Information about the frequency of diabetological check-ups and fundus examinations due to diabetes of each patient was collected from the patient’s medical records and reviewed from the Hospital Information System’s medical records. The authors looked back 12 years when reviewing the Health Insurance Institute’s medical records because the institution has had the Hospital Information System since 2008 with all medical records for each patient attending the ophthalmological and diabetological check-ups and clinical laboratory tests. The personal medical records of patients who came for the first time during the studied period were reviewed.

### 2.2. Metabolic Risk Factors

Body mass index (BMI) was calculated by dividing weight and height squared (kg/m^2^), which were measured using a balance-beam scale and a wall-mounted stadiometer. Systolic blood pressure (SBP) and diastolic blood pressure (DBP) were measured with an appropriate cuff after a 10 min resting period and expressed in mmHg.

Fasting venous blood samples were collected in the morning after an overnight fast to determine metabolic risk factors. Serum lipids were determined using standard enzymatic methods on an automated analyzer (Beckman Coulter AU680, Beckman Coulter, Inc, Brea, CA, USA), while glycated hemoglobin (HbA_1_c) was measured using an automated immunoturbidimetric procedure on a dedicated analyzer (Cobas Integra 400 plus, Roche Ltd., Basel, Switzerland). Renal function was determined using serum creatinine, glomerular filtration rate (GFR), and albumin/creatinine ratio (ACR). Serum creatinine was measured in a fasting blood sample using a routine laboratory method. GFR was estimated using the Chronic Kidney Disease Epidemiology Collaboration (CKD-EPI) formula [12], and a random urine sample was collected to determine the ACR using turbidimetric immunoassay and photometric assays.

### 2.3. Ophthalmologic Retinal Examination

The ophthalmologic retinal examination included indirect slit-lamp fundoscopy, color fundus photography, and optical coherence tomography (OCT) of the macula after mydriasis with eye drops containing 0.5% tropicamide. Color fundus photographs of two fields (macular field, disc/nasal field) of both eyes were taken with the standard 45° fundus camera (Visucam NM/FA, Zeiss, Oberkochen, Germany) according to the EURODIAB retinal photography methodology [13]. Two medical retina specialists independently graded the photographs and assigned a DR level. The final diagnosis for each patient was determined from the level of DR of the worse eye using EURODIAB criteria [13]. Since there was no case where the experts assigned different grades, there was no need for the third grader. OCT of the macula of both eyes was performed using Spectral Domain OCT (SOCT Copernicus REVO, Optopol technology, Raleigh, NC, USA), and DME was defined using the Proposed international clinical diabetic retinopathy and diabetic macular edema disease severity scales [14].

### 2.4. Statistical Analysis

Statistical analysis and graphs were created using StatisticaTM 14.0.1 (TIBCO Software Inc., Palo Alto, CA, USA). The Kolmogorov–Smirnov test was used to test the normality of the data distribution. Results of descriptive analyses were expressed as means ± SD or median (min–max) for continuous variables and numbers (percentages) for categorical variables. Differences between continuous data were determined using one-way ANOVA or Kruskal–Wallis tests (for three groups) and the *t*-test or Mann–Whitney test (for two groups). Nonparametric tests were used when the assumption of homogeneity of variance for tested variables was not met. Multiple comparisons of the Kruskal–Wallis and Scheffe’s post hoc tests were used where needed. The Chi-square test evaluated differences among categorical data. The predictors of DR were identified through binary univariate and multiple logistic regression analyses. The two-way analysis of variance (ANOVA) was used to test the differences in SBP and DBP between the groups according to the DR (no DR, DR) and ACR value (<3.0 mg/mmol, ≥3.0 mg/mmol), and the DR (no DR, DR) and the frequency of fundus examination (once a year, every 3–5 years, 1st fundus examination), and their interactions. The level of statistical significance in each analysis was set at 0.05.

## 3. Results

One hundred and fifty-six T2DM patients (92 males and 64 females) with a mean age of 64.28 ± 7.72 years and a mean diabetes duration of 13.88 ± 7.15 years were included, and their data were analyzed in this cross-sectional study. In this sample, the prevalence of DR was 46.2%. Of those 72 with DR, 44 (61.1%) had nonproliferative DR (NPDR), and 28 (38.9%) had STDR (PDR and/or DME).

Based on their DR status, patients were divided into three groups: no DR (n = 84), NPDR (n = 44), and STDR (n = 28) (Table 1). The three groups did not significantly differ in age, gender, or BMI, but significant differences were observed in the diabetes duration (*p* = 0.015), SBP (*p* < 0.001), DBP (*p* = 0.002), and HbA_1_c (*p* = 0.004). Using the post hoc Scheffe test, patients with STDR had a longer duration of diabetes (*p* = 0.023) and a higher SBP (*p* < 0.001) than those with no DR. However, no significant difference in diabetes duration and SBP was observed between the patients with STDR and NPDR, nor between those with NPDR and no DR. A post hoc Scheffe test for DBP and multiple comparisons for HbA_1_c showed a significant difference between the patients with STDR and those with no DR (DBP, *p* = 0.011; HbA_1_c, *p* = 0.017), as well as between patients with NPDR and those with no DR (DBP, *p* = 0.026; HbA_1_c, *p* = 0.028). The differences in DBP and HbA_1_c among the patients with STDR and NPDR were not statistically significant. No significant differences in lipids and renal function were found between the groups according to the DR status (Table 1).

According to the medical records on the frequency of diabetological check-ups, of all patients, 128 (82.1%) had diabetological check-ups with risk factor testing twice or three times a year, while 28 (17.9%) had it sporadically, once a year, or infrequently. One hundred and thirty-two patients (84.6%) utilized antihypertensive therapy regularly, while 121 patients (77.4%) received hypolipemic treatment, of which 106 patients (66.7%) used statins and 15 patients (9.7%) took fenofibrate.

**Figure 1:**
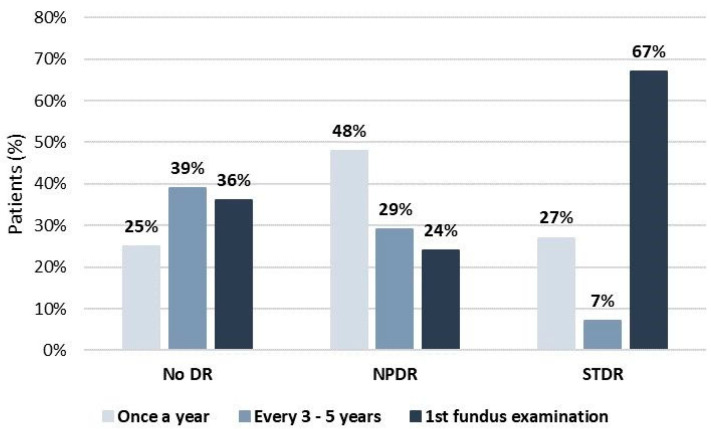
The frequency of fundus examinations in T2DM patients divided into three groups according to the DR status.

Reviewing the medical records on the frequency of fundus examination due to diabetes, 48 (30.8%) of all studied patients performed fundus examination on a regular basis, according to guidelines, once a year, or depending on the DR status; 46 (29.5%) of them performed it irregularly, mostly every 3–5 years; and in 62 (39.7%) patients this was the first fundus examination. Figure 1 presents the frequency of fundus examinations due to diabetes in patients divided into three groups based on their DR status. Patients with no DR mainly did not examine the fundus regularly (n = 63; 75%). Most patients with NPDR (n = 21; 48%) performed a fundus examination on a regular basis, once a year, while in the majority of those with STDR (n = 19; 67%) this was the first fundus examination due to diabetes.

Using the binary logistic regression analysis (no DR/DR), the factors that most strongly predicted DR in T2DM were diabetes duration (*p* = 0.007), HbA_1_c (*p* = 0.006), SBP (*p* < 0.001), and DBP (*p* = 0.002), as shown in Table 2. Even after adjustment for the duration of diabetes and HbA_1_c, the impact of SBP (AOR 1.07, *p* = 0.003) and DBP (AOR 1.13, *p* = 0.005) on the development of DR persisted. A binary logistic regression analysis found no significant associations between DR and the other analyzed variables.

In all the study’s patients, the median/mean of HbA_1_c, SBP, and DBS was 7.0 (5.5–12.1), 138.27 ± 14.13 mmHg, and 82.75 ± 7.50 mmHg, respectively. When dividing the patients into two groups based on the new American Diabetes Association (ADA) blood pressure criteria [15] (Table 3), only 42 T2DM patients had target SBP/DBP < 130/80 mmHg, while in the remaining 114 patients, it was ≥130/80 mmHg. The DR prevalence was higher in patients with higher blood pressure (any DR n = 57; 50%) than in those with target BP (any DR n = 5; 12%) (*p* = 0.043). Among those with higher BP and DR, 30% had NPDR and 20% STDR, while in those with target BP, all 12% had NPDR; none of the patients in this group had STDR. Besides a significantly higher SBP and DBP (*p* < 0.001), T2DM patients with higher BP also had higher BMI scores than those with target blood pressure (*p* = 0.003). Furthermore, ACR was significantly higher in patients with higher BP than in those with target BP (1.4 mg/mmol vs. 0.9 mg/mmol, *p* = 0.033).

**Figure 2:**
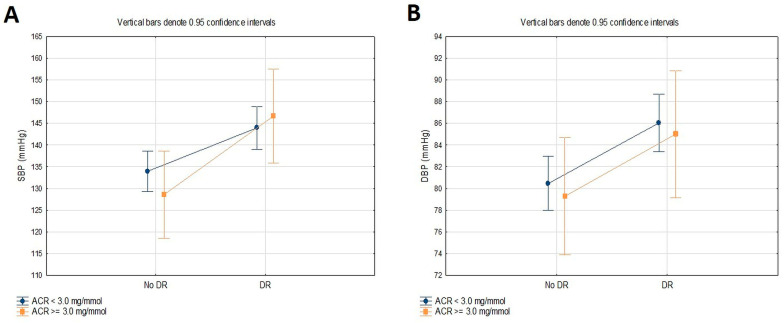
Differences in SBP (**A**) and DBP (**B**) according to the DR and ACR.

Table 4 and Figure 2 present the differences in the SBP and DBP of T2DM patients classified into two groups based on the DR status (no DR, DR) and ACR (<3.0 mg/mmol, ≥3.0 mg/mmol) assessed using ANOVA with two main factors and their interaction. The SBP and DBP were significantly different concerning the presence of DR (*p* < 0.001; *p* = 0.012). However, there were no differences in SBP and DBP concerning the level of ACR, nor in the interaction between the groups of the DR and ACR (*p* > 0.05).

**Figure 3:**
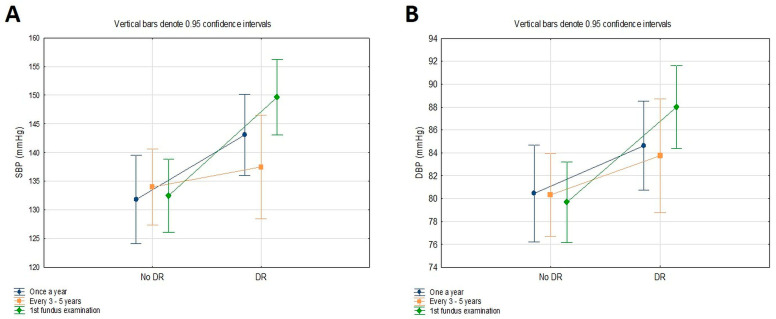
Differences in SBP (**A**) and DBP (**B**) according to the DR status and the frequency of fundus examination.

With a particular emphasis on the two essential analyzed variables, DR and the frequency of fundus examination, Table 5 presents the differences in the SBP and DBP of T2DM patients stratified into two groups based on the DR status (no DR, DR) and the frequency of fundus examination (once a year, every 3–5 years, 1st fundus examination) tested using ANOVA with two main factors and their interaction. The statistically significant differences in SBP and DBP were found based on the DR status (*p* < 0.001; *p* = 0.002), though the differences in the SBP and DBP based on the frequency of fundus examination and the interaction between the two main factors were not significant (*p* > 0.05). Figure 3 graphically presents the differences in SBP and DBP based on the DR status and the frequency of fundus examination. However, the peaks in the SBP (mean 149.67 ± 12.17 mmHg) and DBP (mean 88.00 ± 9.22 mmHg) were observed in T2DM patients with DR and their first fundus examination due to diabetes.

## 4. Discussion

Poor glycemic control, high blood pressure, and hyperlipidemia are the main systemic risk factors associated with DR development and progression, and regular screening for DR is important and well-accepted in many countries in the European region. In our study, patients with no DR mainly did not examine the fundus regularly. Most patients with NPDR underwent a fundus examination regularly, while in the majority of those with STDR, this was the first fundus examination due to diabetes. Furthermore, in our study, most patients (82.1%) have frequent diabetological controls several times a year with HbA_1_c and other metabolic risk factors testing, while the rest (17.9%) have infrequent controls once a year or more rarely. The American Diabetes Association (ADA) guidelines require an HbA_1_c below 7.0% for all nonpregnant persons with diabetes and HbA_1_c testing every six months in those with satisfactory glucose regulation [16]. In our study, patients without DR had the target glucose control with an HbA_1_c of 6.7%. Considering other risk factors, the median SBP in this study was 135 mmHg and the median DBP was 80 mmHg, while serum lipids were mainly satisfactory. Eighty-four percent of all patients in this study received therapy for hypertension and 77% received treatment for hyperlipidemia, of which 67.7% statins and 9.7% fenofibrate. These results are practically consistent with the ADA guidelines for the year 2022 [17] and similar to the results of two large cross-sectional multicenter studies conducted in Europe, CODE-2 and PANORAMA, in which the average values of HbA_1_c in Sweden and the United Kingdom ranged from 7.0% to 7.8% [18,19]. The proportion of patients with an HbA_1_c ≥ 7.0% in the PANORAMA study differed among European countries, from 25.9% in the Netherlands to 52.0% in Turkey. Only 7.5% of all patients included in the PANORAMA study had LDL cholesterol < 2.6 mmol/L, HbA_1_c < 7.0%, and BP < 130/80 mmHg levels [19]. Antihypertensive therapy was taken by 75.3% of patients, and hypolipemic treatment by 65.0%.

Patients with STDR, compared to those with no DR, had a significantly higher SBP. Our study’s main predictors of DR were diabetes duration, HbA_1_c, SBP, and DBP. When dividing the patients into two groups following the new ADA blood pressure criteria, only 42 T2DM patients had the target SBP/DBP < 130/80 mmHg [15]. In addition, none of the patients with SBP/DBP < 130/80 mmHg had STDR. The peaks in SBP and DBP were observed in T2DM patients with DR and their first fundus examination due to diabetes. Besides optimal glucose control, rigorous BP control can also reduce the risk of DR and visual disability in patients with diabetes. The results from the UK Prospective Diabetes Study (UKPDS) suggest that a reduction in SBP by 10 mmHg, independently of HbA_1_c, can decrease the risk of developing microvascular complications by 13% [20,21]. SBP levels are increasing across categories of DR, and a higher SBP is a significant and independent predictor of the presence of DR in normotensive and hypertensive T2DM patients [22]. Although there is still a controversy about whether intensive control of BP will limit the onset and progression of DR, new ADA guidelines for 2024 decreased the target blood pressure control in T2DM to <130/80 mmHg from the previous <140/90 mmHg [15]. This follows the guidelines of BP by the American Heart Association and the American College of Cardiology [23]. A recently published study that included 152,844 patients with diabetes found that a BP over 120/80 mmHg is associated with a significantly increased prevalence of DR by 10–20% in those with and without high BP [24]. In normotensive patients with T2DM, higher SBP is an independent and significant predictor for DR [22]. Considering the strong relationship between DR and hypertension and the two-fold higher risk of hypertension in patients with diabetes compared to nondiabetics, all patients with T2DM should be regularly monitored and treated for hypertension [25].

Almost a third of the world’s population over 18 years has hypertension, defined as an SBP over 140 mmHg. Hypertension induces functional and structural changes in blood vessels, aggravated in patients with diabetes, leading to retinal hemodynamic changes with impaired autoregulation [26]. High systemic BP transmits to the retinal microvasculature and aggravates inflammation and oxidative stress, important underlying conditions involved in the development and progression of DR [27]. High BP also influences the local renin–angiotensin system and increases the angiotensin-II and vascular endothelial growth factor, impairing retina perfusion and facilitating the basement membrane’s thickening, vascular permeability, and neovascularization [28].

Dyslipidemia is also crucial in developing DR and DME, and reducing the response to laser treatment [29]. Treating dyslipidemia with statins and fenofibrate reduces retinal edema and intraretinal hard lipid exudates, and lowers the necessity for laser photocoagulation [30,31]. In this study, most patients received hypolipemic therapy, and although there were slightly higher total and LDL cholesterol levels in the STDR group compared to other groups, there was no significant difference in serum lipids between the studied groups.

In this study, only one-third of patients underwent fundus examinations regularly, once a year, while in most patients with STDR, this was the first fundus examination due to diabetes. A recently published study that included patients with shorter disease duration also found that patients with T2DM at the first ophthalmological examination had the highest percentage of proliferative DR, and over 80% of the patients were symptomatic, complaining of a decrease in visual acuity [32]. These findings emphasize the urgency of early detection and intervention in managing diabetic eye complications in this setting. The peaks in SBP and DBP were observed in T2DM patients with DR at the first fundus examination due to diabetes. Over the past twenty years, in many European countries, different DR screening programs have been developed and introduced in routine diabetes care: hospital-based, regional, and national [33]. The United Kingdom has the largest European national diabetic eye screening program [8]. Denmark is the only EU country with a DR registry (DiaBase) [34]. Ireland, Norway, and Sweden have no DR registry but the basis for the DR screening program is its national diabetic registry for adults and children [9,35,36]. Nowadays, fundus examination has been obtained with new technologies in ophthalmology, such as different fundus cameras and portable screening devices [37]. These methods can involve a much wider group of healthcare professionals, such as nurses and optometrists, to take fundus images and send them to an ophthalmologist for interpretation or even to automated artificial intelligence (AI)-based grading systems for fast, automated image analysis [38,39,40]. New DR screening methods, compared with the traditional retina examination, can cover a larger population of patients with diabetes, simultaneously saving other resources such as time and money [41]. The first AI-based ophthalmological tool approved by the FDA for the assessment of more than mild DR has a high sensitivity of 87% and specificity of 91%. DR screening with AI significantly increases the availability of ophthalmological examination considering the limited number of ophthalmologists [42]. Patients with no or mild DR are suggested to undertake rescreening in a year without needing an ophthalmologist examination. Only those patients with moderate or severe NPDR, PDR, and DME are referred to ophthalmologists.

Several limitations of this study should be addressed. First, the lack of a national registry and reliance on a single center may likely underestimate the true rate of prior eye examinations, affecting the study’s validity. Second, the small sample size and limited scope limit the generalizability of the findings to larger populations and necessitate careful consideration of its findings in broader policy discussions. Third, our study included only the Caucasian race. Fourth, we used an office BP measurement when an ambulatory BP measurement is more valuable and accurate. Fifth, since we included only patients referred to our hospital, the Diabetes Referral Centre in Croatia, selection bias must be considered.

## 5. Conclusions

In this studied sample, DR prevalence was very high and strongly related to blood pressure and a lack of regular fundus examination. However, the SBP and DBP were significantly higher in patients with STDR than in those without DR. None of the patients with target BP (<130/80 mmHg) had STDR. Only one-third of patients underwent fundus examinations regularly, once a year, while in most patients with STDR, this was the first fundus examination due to diabetes. These results emphasize the necessity of establishing systematic DR screening in routine diabetes care and targeting blood pressure levels according to T2DM guidelines. Several methodological and interpretative considerations of our study warrant attention, and a larger systematic study should explore the implications of the findings in a broader cross-section of the Croatian population.

## Figures and Tables

**Table 1:** Basic and clinical characteristics, metabolic risk factors, and renal function of T2DM patients (n = 156) divided into three groups according to the DR status.

	No DR (n = 84)	NPDR (n = 44)	STDR (n = 28)	*p*-Value
Age (yr)	65.53 ± 7.52	64.68 ± 7.23	63.97 ± 7.74	0.139
Gender (m/f) (%)	52/48	59/41	68/32	0.195
Diabetes duration (yr)	11.83 ± 6.77	15.28 ± 5.44	17.03 ± 4.28	0.015
BMI (kg/m^2^)	29.59 ± 4.70	31.39 ± 5.38	28.21 ± 2.49	0.119
SBP (mmHg)	132.85 ± 11.21	140.45 ± 15.58	151.07 ± 10.77	<0.001
DBP (mmHg)	80.12 ± 5.24	85.23 ± 9.69	86.79 ± 6.68	0.002
HbA_1_c (%)	6.7 (5.5–9.4)	7.3 (5.8–9.7)	7.9 (5.9–12.2)	0.004
Total cholesterol (mmol/L)	4.7 (2.7–10.2)	4.6 (3.1–6.3)	5.3 (2.7–7.1)	0.587
HDL cholesterol (mmol/L)	1.4 (0.8–2.6)	1.2 (0.8–2.1)	1.2 (1.0–2.0)	0.258
LDL cholesterol (mmol/L)	2.5 (1.1–7.1)	2.4 (0.9–4.3)	2.9 (1.3–4.9)	0.328
Triglycerides (mmol/L)	1.6 (0.5–6.3)	1.9 (0.8–7.0)	1.3 (0.5–3.4)	0.099
Serum creatinine (μmol/L)	73.5 (50–130)	77.5 (49–135)	74 (42–156)	0.680
eGFR (mL/min/1.73 m^2^)	85.5 (43–108)	82 (35–105)	89.5 (38–108)	0.412
ACR (mg/mmol)	1.4 (0.3–9.0)	1.3 (0.5–18.1)	1.1 (0.4–19.1)	0.772

Legend: Values are means ± SD, percentages, or medians (min–max). *p*-values for comparison between patients with different levels of DR. BMI, body mass index; SBP, systolic blood pressure; DBP, diastolic blood pressure; HbA_1_c, glycated hemoglobin; HDL, high-density lipoprotein cholesterol; LDL, low-density lipoprotein cholesterol; eGFR, estimated glomerular filtration rate; ACR, albumin/creatinine ratio.

**Table 2:** Risk factors and predictors for development of diabetic retinopathy as a dichotomous variable in T2DM using univariate and multiple logistic regression analysis.

Variable	OR (95% CI)	*p*-Value	AOR (95% CI) *	*p*-Value *
Diabetes duration	1.11 (1.05–1.21)	0.007	/	
HbA_1_c	1.98 (1.20–3.27)	0.006	/	
SBP	1.07 (1.02–1.12)	<0.001	1.07 (1.02–1.12)	0.003
DBP	1.14 (1.05–1.23)	0.002	1.13 (1.04–1.24)	0.005

* OR after adjustment for diabetes duration and HbA_1_c; Legend: HbA_1_c, glycated hemoglobin; SBP, systolic blood pressure; DBP, diastolic blood pressure.

**Table 3:** Basic and clinical characteristics, metabolic risk factors, renal function, and DR status of T2DM patients (n = 156) divided into two groups according to blood pressure.

	<130/80 mmHg (n = 42)	≥130/80 mmHg (n = 114)	*p*-Value
DR status (No DR/DR) (%)	88/12	50/50	0.043
Age (yr)	63.37 ± 10.79	64.31 ± 7.38	0.746
Gender (m/f) (%)	87/13	56/44	0.083
Diabetes duration (yr)	9.50 ± 7.80	14.38 ± 6.96	0.067
BMI (kg/m^2^)	25.36 ± 3.03	30.37 ± 4.57	0.003
SBP (mmHg)	120.62 ± 6.78	140.28 ± 13.35	<0.001
DBP (mmHg)	73.12 ± 4.58	84.86 ± 3.97	<0.001
HbA_1_c (%)	6.6 (5.6–9.1)	7.1 (5.5–12.1)	0.553
Total cholesterol (mmol/L)	4.5 (3.8–7.4)	4.6 (2.7–10.2)	0.680
HDL cholesterol (mmol/L)	1.4 (1.2–1.9)	1.3 (0.8–2.5)	0.429
LDL cholesterol (mmol/L)	2.5 (1.6–4.7)	2.4 (0.9–7.1)	0.415
Triglycerides (mmol/L)	1.3 (0.5–2.7)	1.6 (0.5–7.0)	0.323
Serum creatinine (μmol/L)	79.0 (56–90)	74.5 (42–156)	0.993
eGFR (mL/min/1.73 m^2^)	96.0 (68–104)	85.5 (35–119)	0.202
ACR (mg/mmol)	0.9 (0.3–6.4)	1.4 (0.4–19.1)	0.033

Legend: Values are means ± SD, percentages, or medians (min–max). *p*-values for comparison between patients with different levels of blood pressure. BMI, body mass index; SBP, systolic blood pressure; DBP, diastolic blood pressure; HbA_1_c, glycated hemoglobin; HDL, high-density lipoprotein cholesterol; LDL, low-density lipoprotein cholesterol; eGFR, estimated glomerular filtration rate; ACR, albumin/creatinine ratio.

**Table 4:** Results of two-way ANOVA for the differences in SBP and DBP according to the DR status, ACR, and their interaction.

		SBP			DBP	
	df	F	*p*	df	F	*p*
DR	1	12.023	<0.001	1	6.613	0.012
ACR	1	0.108	0.743	1	0.252	0.617
DR and ACR	1	0.990	0.323	1	0.001	0.975

Legend: SBP, systolic blood pressure; DBP, diastolic blood pressure; DR, diabetic retinopathy; ACR, albumin/creatinine ratio.

**Table 5:** Results of two-way ANOVA for the differences in SBP and DBP according to the DR status, the frequency of fundus examination, and their interaction.

		SBP			DBP	
	df	F	*p*	df	F	*p*
DR	1	12.647	<0.001	1	10.428	0.002
Frequency of fundus examination	1	1.185	0.312	1	0.467	0.630
DR and frequency of fundus examination	1	1.767	0.178	1	0.952	0.392

Legend: SBP, systolic blood pressure; DBP, diastolic blood pressure; DR, diabetic retinopathy.

## Data Availability

The data presented in this study are available upon specific request from the corresponding author.
